# Angiotensin II Type 1 Receptor Blocker Usage Prevents Oxidative Stress and Muscle Dysfunction in HIV

**DOI:** 10.1111/fcp.70016

**Published:** 2025-05-27

**Authors:** Rafael Deminice, Paola Sanches Cella, Ana Lúcia Borsari, Camila S. Padilha, Vitor Hugo Fernando de Oliveira

**Affiliations:** ^1^ Physical Education and Sports Institute State University of Londrina Londrina Brazil; ^2^ Biology of Ageing Laboratory, Centre for Healthy Ageing, Centenary Institute of Cancer Medicine and Cell Biology Royal Prince Alfred Hospital Sidney NSW Australia; ^3^ Faculty of Medicine and Health, Charles Perkins Centre The University of Sydney Sydney NSW Australia; ^4^ Faculty of Health, School of Sport, Exercise and Rehabilitation Sciences University of Technology Sydney Ultimo NSW Australia; ^5^ Department of Child, Family and Population Health Nursing University of Washington Seattle Washington USA

**Keywords:** HIV, hypertension, renin‐angiotensin system, sarcopenia

## Abstract

**Background:**

We aimed to elucidate the role of Angiotensin II type 1 receptor (AT1R) blocker usage in muscle wasting and dysfunction related to HIV.

**Research Design and Methods:**

Appendicular skeletal muscle mass, higher and lower limb strength, and physical fitness were determined in people living with HIV (PWH) using AT1R blockers users (*n* = 33), angiotensin‐converting enzyme (ACE) inhibitors (*n* = 28), or not using antihypertensive drugs (*n* = 33). Groups had similar age, sex, race, BMI, and time of HIV infection. Muscle biopsies were performed to determine the abundance of AT1R, the relative abundance of selected proteins related to proteolysis, antioxidant enzymes, and oxidative stress. Plasma angiotensin II, IL‐6, and TNF‐alpha were also determined.

**Results:**

PWH using AT1R blocker presented higher strength, physical fitness, and muscle mass than PWH using ACE inhibitors or not using antihypertensive drugs. Although both PWH using AT1R blockers and ACE inhibitors presented reduced angiotensin II plasma levels, only PWH using AT1R blockers presented lower skeletal muscle AT1R activation, lower plasma oxidative stress markers, lower skeletal muscle oxidative stress (4‐HNE), and proteolysis markers (Atrogin‐1, Murf‐1).

**Conclusion:**

AT1R blocker usage protects against oxidative stress and activated proteolysis, contributing to the prevention of muscle wasting and dysfunction among PWH.

## 1 Introduction

Sarcopenia is a musculoskeletal disease characterized by progressive and generalized loss of muscle strength associated with low muscle quantity and quality [1]. Loss of muscle strength is associated with adverse outcomes, including falls, morbidities, and higher mortality rates in the general population [2]. Due to the intersecting effects of aging, human immunodeficiency virus (HIV), antiretroviral therapy (ART) toxicity, and some lifestyle and sociodemographic risk factors such as tobacco, substance abuse, food insecurity, and physical inactivity, people with HIV (PWH) are highly susceptible to sarcopenia development [3]. A recent meta‐analysis from our group demonstrated a sarcopenia prevalence of 24.1% among PWH and a 6.1 greater odds of sarcopenia when compared to people without HIV [4].

Emerging evidence reveals that hyperactivity of the renin‐angiotensin system (RAS), a master blood pressure regulator contributes to skeletal muscle wasting [5]. Indeed, elevated blood circulating angiotensin II (Ang‐II) and RAS activation trigger several signaling pathways involved in muscle physiology (for a comprehensive review of the role of RAS in skeletal muscle physiology, see [5]. Briefly, elevated Ang‐II plasma levels can contribute to skeletal muscle atrophy through indirect and direct mechanisms. Indirectly, Ang‐II can stimulate the release of circulating interleukins like interleukin‐6 (IL‐6) and tumor necrosis factor‐alpha (TNF‐α) [6]. These factors play significant roles in upregulating protein degradation via the nuclear factor kappa‐B (NFκ‐B) pathway, resulting in wasting muscle [7]. Directly, Ang‐II interacts with angiotensin II type I receptors (AT1Rs) on the sarcolemma of muscle fibers to increase reactive oxygen species (ROS) formation [8, 9] that upregulate the ubiquitin‐proteasome system (UPS) pathway, resulting in muscle atrophy and depressing the anabolic response [10, 11]. Importantly, we recently demonstrated that AT1Rs are present in both human and rat skeletal muscle fibers, providing the foundation that elevated Ang‐II can induce skeletal muscle atrophy [12].

Despite all the premise and relevant preclinical data demonstrating that elevated Ang‐II and RAS activation play a key role in skeletal muscle atrophy, whether RAS plays a role in skeletal muscle mass and function in humans with HIV is currently unknown. This knowledge is relevant because PWH are highly susceptible to muscle dysfunctions. In addition, hypertension is independently and highly prevalent among PWH [13], so the use of AT1R blockers (e.g., losartan) and angiotensin‐converting enzyme (ACE) inhibitors (e.g., enalapril) is common among this population. Thus, we aimed to elucidate the role of AT1R blocker usage in muscle wasting and dysfunction related to HIV. We hypothesized that RAS plays a key role in the skeletal muscle health of PWH and that AT1R blocker usage prevents muscle wasting and dysfunction related to HIV.

## 2 Patients and Methods

### 2.1 Study Design

This is a descriptive cross‐sectional study that utilized data from a larger investigation aiming at assessing functional, morphological, and molecular characteristics of sarcopenia in PWH. The data were collected between April 2018 and December 2019. The participants initially underwent an interview regarding sociodemographic and medical information during their regular medical appointments at their HIV health service. Measurements of body mass, height, appendicular skeletal muscle mass (ASMM), handgrip strength, and physical function were collected. Participants' medical history was also extracted through chart review at the end of the visit. After this initial visit, the participants were invited to perform additional assessments, which included the blood draw and muscle biopsy analyzed in this study.

### 2.2 Participants

PWH from the Londrina State University Hospital and the Integrated Center for Infectious Diseases of Londrina in south Brazil were invited to participate in this study. Inclusion criteria were as follows: (1) having a confirmed HIV‐1 diagnosis noted in their medical records, (2) ≥ 18 years of age, (3) being prescribed ART, (4) not being on hormone replacement therapy or using anabolic drugs, (5) not having an advanced immunodeficiency status or active opportunistic infection, and (6) having the cognitive and physical capacity to perform the tests. Exclusion criteria were as follows: (1) PWH using AT1R blockers or ACE inhibitors if the use was introduced for less than 6 months and (2) PWH using antihypertensive drugs other than AT1R blockers and ACE inhibitors, such as beta‐blockers.

All participants were informed about the study procedures and signed an informed consent form before any procedure. The study protocol was approved by the Ethics Board Committee for Research Involving Human Subjects of Londrina State University (#2.305.624) and followed the ethical guidelines of the Helsinki Declaration and Brazilian law. All study participants were volunteers and did not receive any kind of compensation.

**FIGURE 1:**
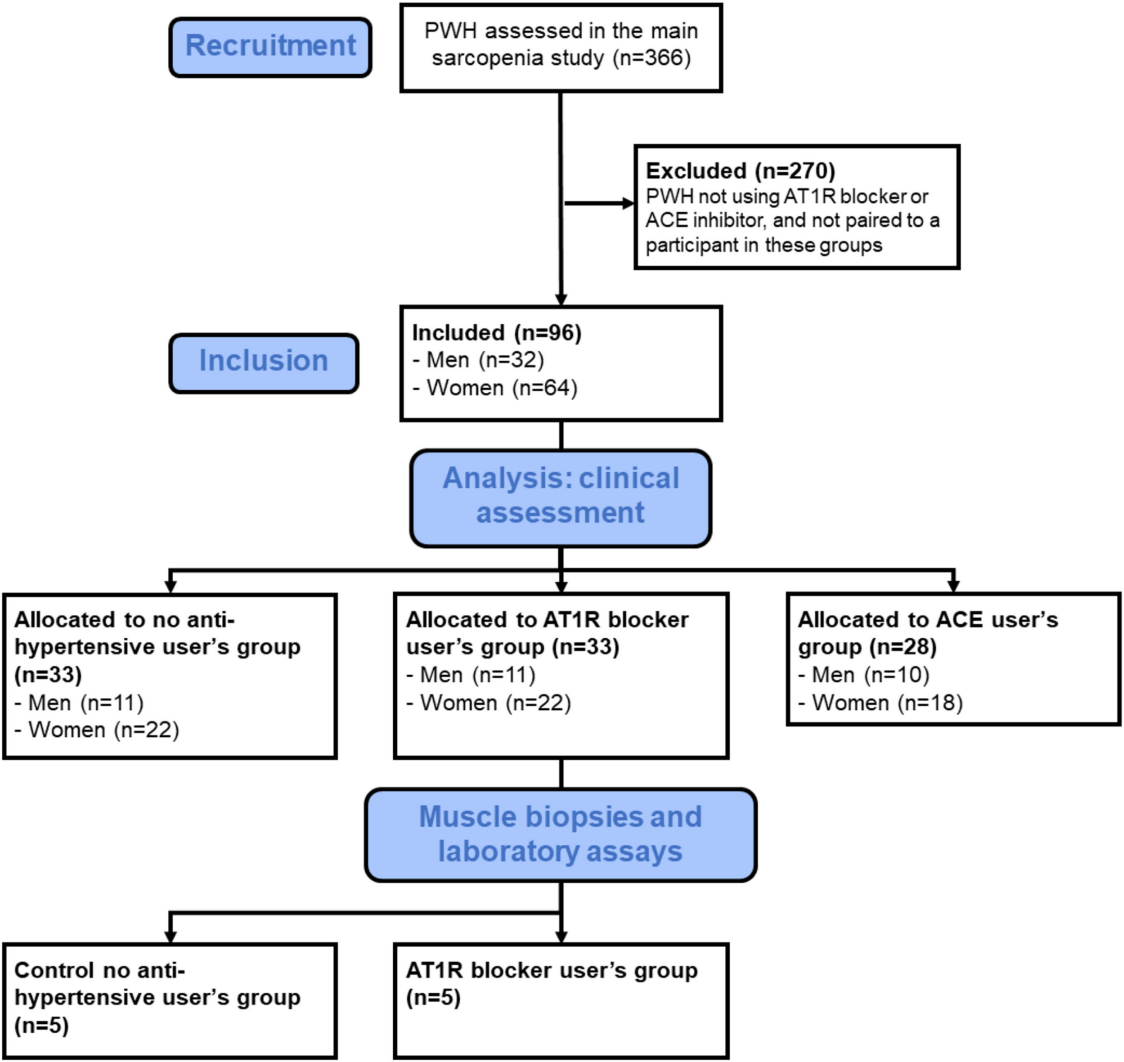
STROBE flow chart. STROBE, Strengthening the Reporting of Observational Studies in Epidemiology.

The primary study evaluated 366 PWH (189 men and 177 women) with an average age of 47.9 ± 11.9 years. Among them, 78 were hypertensive and using some antihypertensive drug (33 AT1R blocker users, 28 ACE blocker users, and 18 beta‐blocker users). The AT1R blocker users (average of 38.5 ± 15.5 months of antihypertensive drug use) were initially identified, and a posteriori paired by age (±1 year), sex, body mass index (BMI, ±1 kg/m2), and race with PWH who were not using any antihypertensive drug. Additionally, the 28 PWH using ACE blockers (average of 34.7 ± 19.2 months of antihypertensive drug use) were included in the study as a positive control group. Although not pared, ACE users had similar characteristics to the other two groups (see Table 1). Therefore, this study included a sample of 96 participants, comprising 33 AT1R blocker users, 28 ACE blocker users, and 33 nonantihypertensive drug users. These individuals were assessed for clinical measures of muscle mass and function. Beta‐blocker users were excluded because it has no direct relation with RAS metabolism. For blood markers and muscle biopsy sampling, 45 participants (18 PWH not using antihypertensive drugs, 14 using AT1R blockers, and 13 using ACE inhibitors) consented to provide blood samples, and 10 participants (5 using AT1R blockers and 5 not using antihypertensive drugs) agreed to undergo muscle biopsy. The flow of participants is illustrated in Figure 1.

**TABLE 1:** Participants characteristics.

	PWH not using antihypertensive drugs (*n* = 33)	PHW using AT1R blockers (*n* = 33)	PWH using ACE inhibitors (*n* = 28)	*p*‐value
Age (years)	53.2 ± 7.8	53.1 ± 8.0	54.1 ± 8.6	0.87
Body mass index (kg/m ^2^ )	29.3 ± 4.1	30.8 ± 6.1	29.2 ± 8.7	0.54
Male	11 (33)	11 (33)	10 (36)	0.84
Race				0.52
Black or Black mixed	13 (39)	13 (39)	12 (43)	
White, Asian, or Brazilian Indian	20 (61)	20 (61)	16 (57)	
Systolic blood pressure (mmHg)	119.1 ± 18.2	127.3 ± 16.6	124.2 ± 14.2	0.13
Diastolic blood pressure (mmHg)	79.7 ± 9.5	85.2 ± 9.7	81.5 ± 10.7	0.09
Time living with HIV (years)	11.4 ± 6.7	10.8 ± 8.4	11.7 ± 6.6	0.88
Time of ART use (years)	9.7 ± 6.6	8.8 ± 8.1	10.6 ± 6.6	0.61
CD4 + lymphocytes				0.48
≤ 500 cells/mm ^3^	15 (45)	14 (42)	13 (46)	
>500 cells/mm ^3^	18 (55)	19 (58)	15 (54)	
CD8 + lymphocytes (cells/mm ^3^ )	1024.2 ± 457.8	1111.4 ± 57.5	1084.5 ± 475.9	0.77
CD4 + nadir				0.54
≤ 200 cells/mm ^3^	12 (36)	12 (36)	11 (39)	
>200 cells/mm ^3^	21 (64)	21 (64)	17 (61)	
HIV viral load (copies/mm ^3)^				0.18
< 40 (undetectable)	26 (78)	27 (81)	24 (86)	
≥ 40 (detectable)	7 (22)	6 (19)	4 (14)	
ART regimen composition				0.34
NRTI + PI	14 (42)	16 (49)	12 (43)	
NRTI + NNRTI	10 (31)	9 (27)	8 (29)	
NRTI + II	5 (15)	4 (12)	4 (14)	
NRTI + PI + II	1 (3)	2 (6)	2 (7)	
Other	3 (9)	2 (6)	2 (7)	
Antihypertensive drug usage				
Losartan	—	32 (97)	—	
Valsartan	—	1 (3)	—	
Enalapril	—	—	18 (64)	
Captopril	—	—	8 (29)	
Ramipril	—	—	2 (7)	
Time of antihypertensive drug use (months)	—	38.5 ± 15.5	34.7 ± 19.2	0.42

*Note:* Continuous variables are expressed as mean ± SD, and categorical variables are expressed as absolute (relative) values.

Abbreviations: ART, HIV antiretroviral therapy; II, integrase inhibitor; NNRTI, nonnucleoside reverse transcriptase inhibitor; NRTI, nucleoside reverse transcriptase inhibitor; PI, protease inhibitor.

### 2.3 Demographic and Clinical Characteristics

Information on race and medical history was collected through interviews using a questionnaire. Additional medical information was obtained from the patient's medical records, including (a) most recent blood pressure, T‐CD4+, and T‐CD8 + lymphocytes; (b) duration and type of antihypertensive drug use; and (c) current use and total duration of individual ART drugs and drug classes.

### 2.4 Muscle Strength, Muscle Mass, Physical Function, and Sarcopenia Determination

The measures and cutoffs for muscle strength, muscle mass, and physical function were selected following the revised sarcopenia definition and diagnosis from the European Working Group on Sarcopenia in Older People [1]. Handgrip strength was measured using a Jamar dynamometer [1]. To measure ASMM, we utilized a bioelectrical impedance device (Bia Analyzer, manufactured by Rushford NanoElectroChemistry Co., Rushford, MN, USA) as described by Kyle et al. [14]. ASMM was then divided by the squared height of the participant, resulting in the appendicular skeletal muscle mass index (ASMM/m^2^) [1]. To evaluate the physical function, we used the Short Physical Performance Battery (SPPB) [15], which comprises the following three different tests: (1) static balance, (2) five times sit‐to‐stand chair test, and (3) gait speed test.

Sarcopenia was determined according to the Cruz‐Jentoft et al. [1]: (1) no sarcopenia (normal muscle strength), (2) probable sarcopenia (low muscle strength), (3) sarcopenia (low muscle strength plus low muscle mass), and (4) severe sarcopenia (low muscle strength, low muscle mass, and low physical function). Low muscle strength occurred when the chair stand test performance was > 15 s for both sexes. Low muscle mass occurred when ASMM/m^2^ was < 7.0 kg/m^2^ for men and < 5.5 kg/m^2^ for women, or ASM was <20 kg for men and <15 kg for women. Low physical function occurred when SPPB was ≤ 8 points score, or gait speed was ≤ 0.8 m/s.

### 2.5 Angiotensin, Cytokines, and Oxidative Stress Analysis

Peripheral blood samples (~5 mL) were drawn from the cubital vein in EDTA containing tubes and centrifuged at 1000 *g* for 10 min at 4°C for plasma acquisition. Plasma concentrations of Ang‐II were determined using an Enzyme Immunoassay (EIA) kit from Sigma (catalog #RAB0010‐1KT, St. Louis, MO, USA). Angiotensin 1–7 (Catalog #EH2583, FineTest, Bolder, CO, USA) and interleukins necrosis factor‐alpha (TNF‐α, Ref: #88‐7340‐88), and interleukin 6 (IL‐6, Ref: #88‐7064‐88) were determined using the enzyme‐linked immunosorbent assay (ELISA) Ready‐SET‐Go kit from eBioscience (San Diego, CA, USA). The concentrations of malondialdehyde (MDA) and advanced oxidation protein products (AOPP) were determined as previously described by Spirlandely et al. [16] and Witko‐Sarsat et al. [17], respectively.

### 2.6 Skeletal Muscle Biopsy and Analysis

*Vastus lateralis* skeletal muscle samples (~150–200 mg) were obtained from 10 participants (five participants from AT1R blockers users and five from no antihypertensive drugs users). Half of the obtained muscle tissue was quickly frozen for immunoblotting analysis, and a portion of the muscle tissue was used for microscopy analysis.

#### 2.6.1 Immunoblotting Analysis

Proteins extraction from muscle samples and immunoblotting preparation was performed as described previously by Deminice et al. [12]. The membranes were incubated with the primary antibody in 5% defatted bovine serum albumin in TBS‐T (anti‐AT1R dilution 1:1000 catalog #AB15552, anti‐Fbx32/atrogin‐1 dilution 1:1000 catalog #ab74023, anti‐MuRF‐1 dilution 1:1000 catalog #ab172479; anti‐4HNE dilution 1:1000 catalog #AB5605). Band quantification was performed by optical densitometry using Image Studio Lite Ver 5.2 (Li‐Cor Biosciences, Lincoln, NE).

#### 2.6.2 Cross‐Section Area (CSA) Analysis

Fresh muscle tissue was fixed in 4% formaldehyde for 24 h, dehydrated with graded ethanol, and embedded in paraffin blocks following routine procedures. Semithin sections (5 μm) were cut in a microtome, applied on silane‐coated slides, deparaffinized, and stained with hematoxylin and eosin (H&E). Images were captured with an optical microscope at a magnification of 200x, and the CSA of muscle fibers was quantified in five participants of each group, with approximately six images and 180 fibers per participant, using ImageJ software (National Institutes of Health, Bethesda, MD).

### 2.7 Statistical Analysis

Descriptive statistics were used to present continuous variables as mean ± standard deviation (SD) for parametric distribution data and median and quartiles for nonparametric distribution data. Categorical variables were presented as prevalence (percentage). The Shapiro–Wilk test was applied to test for data normality, and Levene's test was used to analyze the homogeneity of variances. For parametric data, one‐way ANOVAs with Tukey post‐test and Student *t*‐test were used to examine differences between groups, while the Mann–Whitney test was used for nonparametric data. To determine differences in contingency tables, the Chi‐square test was employed. A *p*‐value of ≤ 0.05 was considered statistically significant.

## 3 Results

Table 1 presents the characteristics of the study participants. There were no statistically significant differences (*p* > 0.05) between groups concerning age, sex, race, BMI, blood pressure, and HIV‐related factors, including time living with HIV, ART regimen, CD4, and CD8 cell counts (Table 1). Losartan was the most commonly used AT1R blocker (97%), followed by Valsartan (3%). Among ACE inhibitors, enalapril (64%) was the most prevalent, followed by Captopril (29%) and Ramipril (7%).

**FIGURE 2:**
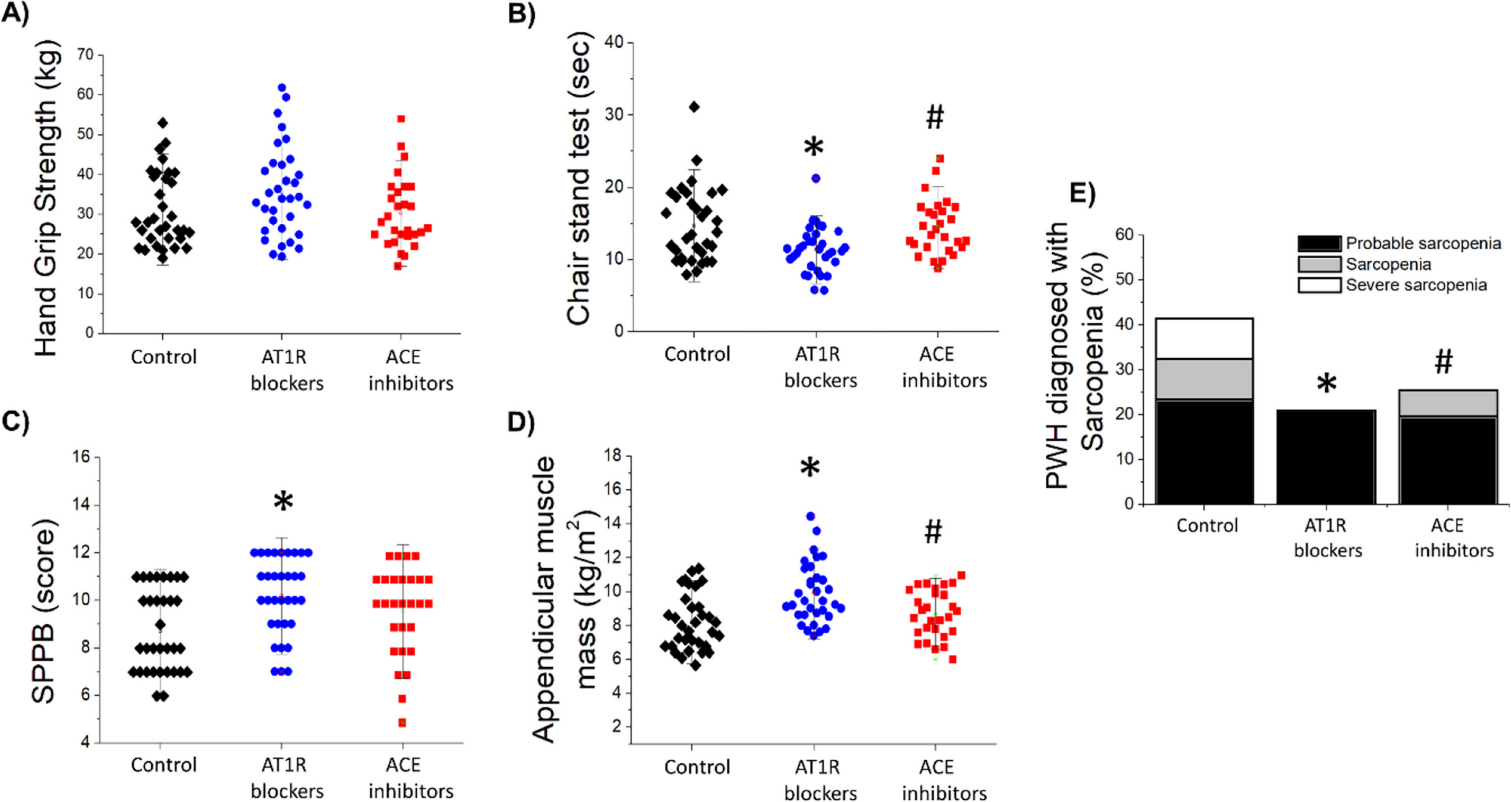
Comparison of hand grip strength (A), chair stand test (B), short physical performance battery test score (C), appendicular muscle mass index (D), and sarcopenia prevalence (E) between people with HIV not using antihypertensive drugs (control), using Ang‐II type 1 receptor blockers (AT1R blockers) and using angiotensin‐converting enzyme inhibitors users (ACE blockers). Data are median, quartile interval, and individual data. * Indicates significant differences from PWH not using antihypertensive drugs (control) and # from PWH using AT1R blockers by ANOVA one‐way followed by Tukey post‐test.

The results in Figure 2 showed that PWH using AT1R blockers exhibited higher lower limb muscle strength as demonstrated by the chair stand test when compared to PWH not using any antihypertensive drugs or ACE inhibitor users (Figure 2B). Additionally, ASMM and physical function measured by the SPPB score were also higher in PWH using AT1R blockers when compared to PWH not using any antihypertensive drugs or ACE inhibitor users (Figure 2C,D). Moreover, the prevalence of sarcopenia and severe sarcopenia was lower among PWH using AT1R blockers compared to the other groups (Figure 2E). No differences were observed in hand grip strength between the groups (Figure 2A).

**FIGURE 3:**
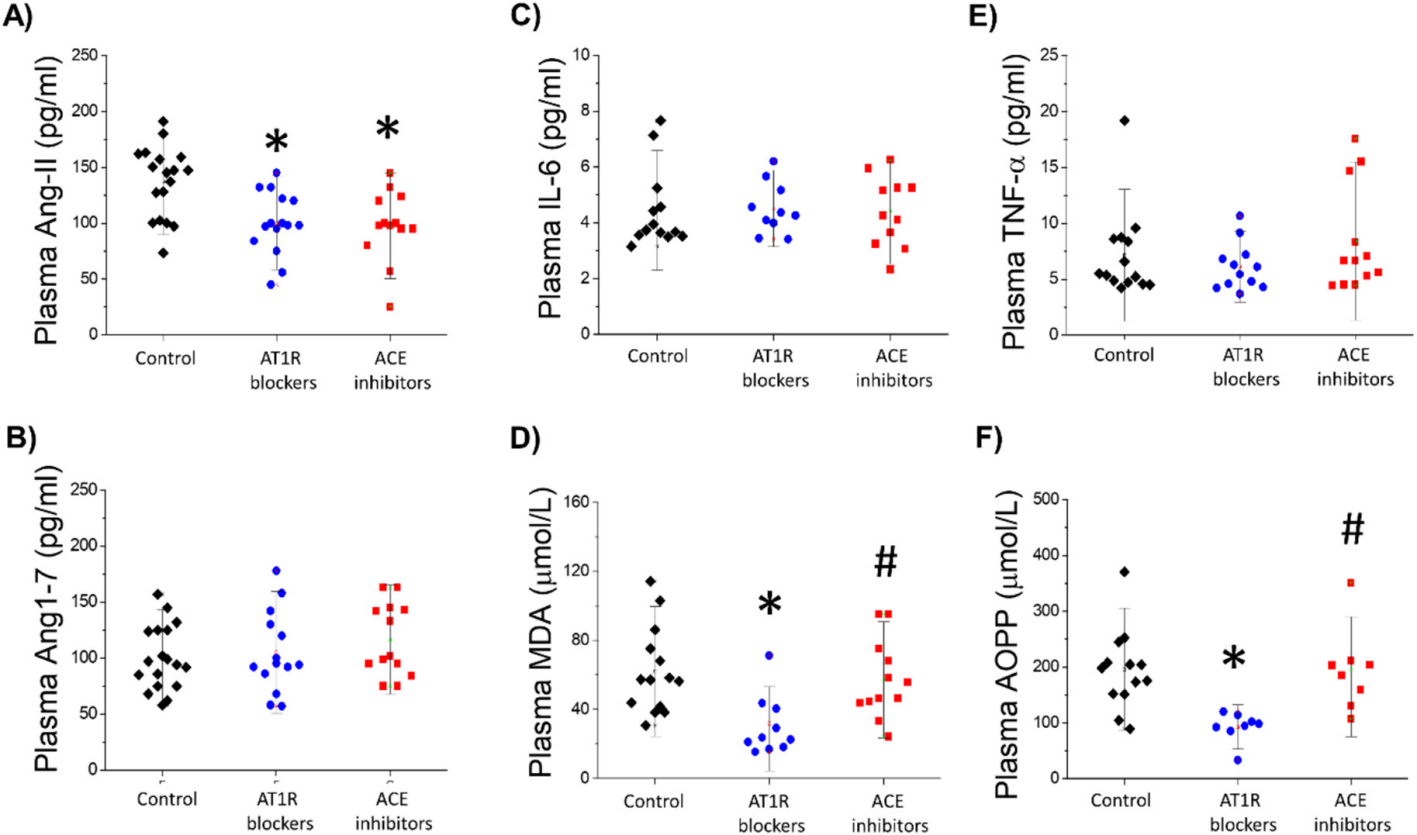
Comparison between plasma levels of (A) angiotensin II, (B) interleukin‐6, (C) tumor necrosis factor‐alpha, (D) malondialdehyde and (E) advanced oxidation protein products between PWH not using antihypertensive drugs (Control), PWH using Ang‐II type 1 receptor blockers (AT1R blockers), and PWH using angiotensin‐converting enzyme inhibitors users (ACE blockers). Data are median, quartile interval, and individual data. * Indicates significant differences from PWH not using antihypertensive drugs (control) and # from PWH using AT1R blockers by ANOVA one‐way followed by Tukey post‐test.

Figure 3 demonstrated that both PWH using AT1R blockers or ACE inhibitors presented lower Ang‐II plasma levels compared to PWH not using antihypertensive drugs (Figure 3A), but no differences between groups were demonstrated for Ang1–7 plasma concentration (Figure 3B). In addition, only PWH using AT1R blockers presented reduced plasma lipid and protein oxidation markers when compared to both PWH using ACE inhibitors and PWH not using antihypertensive drugs (Figure 3D,F). No differences in plasma IL‐6 and TNF‐alpha were demonstrated between groups (Figure 3C,E).

**FIGURE 4:**
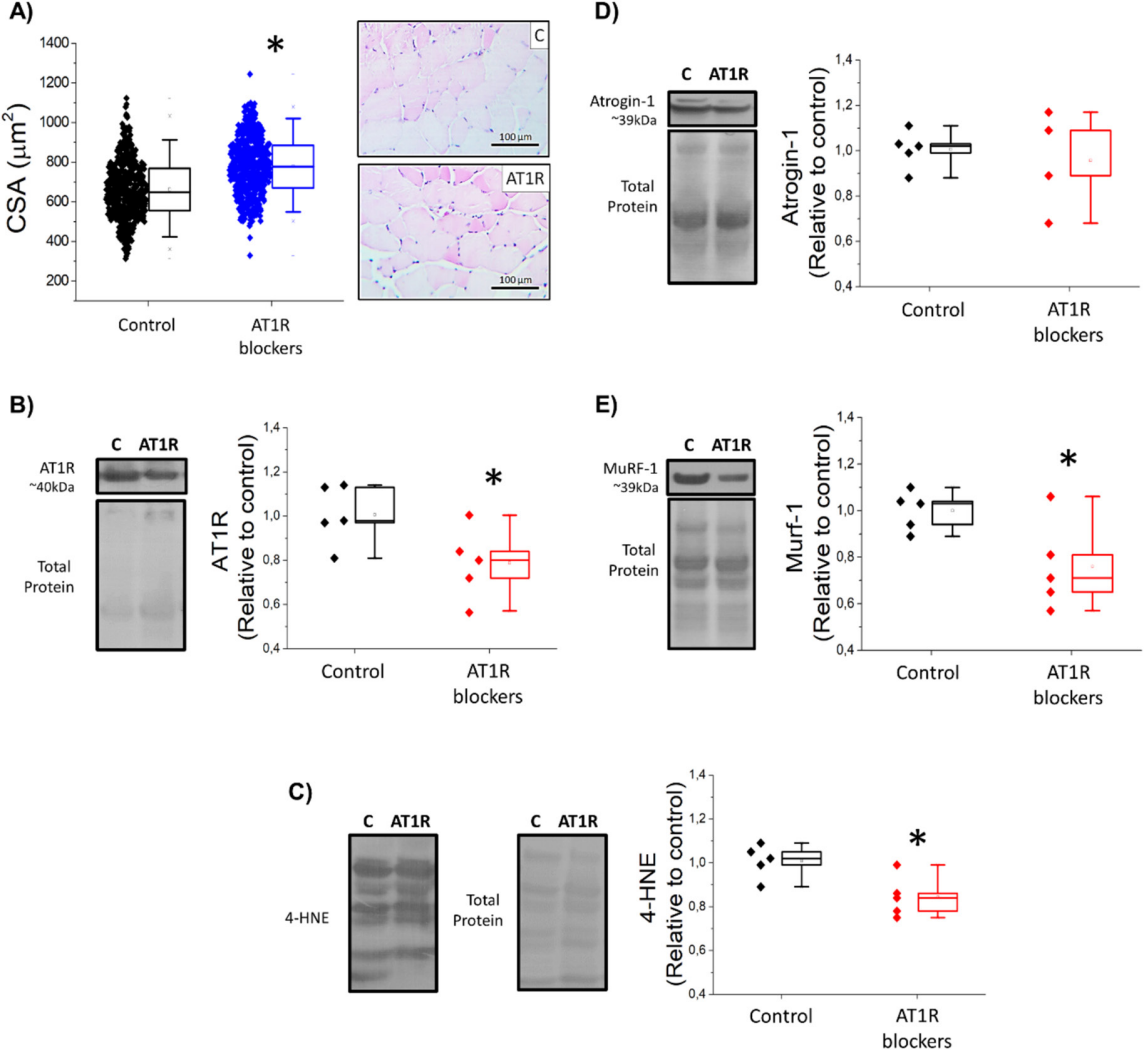
Comparison of skeletal muscle (A) cross‐sectional area and skeletal muscle protein abundance of (B) Ang‐II type 1 receptors, (C) 4‐hydroxynonenal, and (D) MuRF‐1 and (E) atrogin‐1 between PWH not using antihypertensive drugs (control), PWH using Ang‐II type 1 receptor blockers (AT1R blockers) and PWH using angiotensin‐converting enzyme inhibitors users (ACE blockers). Data are median, quartile interval, and individual data. * Indicates significant differences from PWH not using antihypertensive drugs (control) by Mann–Whitney test (panel A) and Student *t*‐test (panels B to E).

Muscle biopsies revealed that PWH using AT1R blockers presented higher skeletal myofiber CSA (Control 657, 552‐784 AT1R 768, 689‐889, μm^2^) and reduced AT1R abundance (Figure 4A,B) compared to PWH not using antihypertensive drugs. PWH using AT1R blockers also had lower oxidative stress and lower proteolysis markers all compared to PWH not using antihypertensive drugs (Figure 4).

## 4 Discussion

Our findings provide novel information about the role that Ang‐II/AT1R plays in the skeletal muscle of PWH. First, we demonstrated that PWH using AT1R blockers present higher lower limb muscle strength (chair stand test) and muscle mass and are less likely to develop sarcopenia. Second, our data identify that PWH using AT1R blockers but not ACE inhibitors are less prone to systemic and skeletal muscle oxidative stress response, as well as muscle atrophy and protein degradation. These findings are important because we confirmed our initial hypothesis that RAS plays a key role in the skeletal muscle health of PWH. In addition, AT1R blocker usage may have a preventive role on muscle wasting and dysfunction related to HIV via a direct mechanism of Ang‐II/AT1R (ROS production and E3 ubiquitin ligases). This suggests that AT1R activation by Ang‐II is a key element in the sarcopenia development in PWH. To our knowledge, this is the first human study demonstrating that RAS plays a key role in skeletal muscle health and AT1R blockers such as losartan can protect PWH from sarcopenia development.

While elevated levels of Ang‐II are linked to various harmful health conditions, the interaction between Ang‐II and the AT1R in muscle wasting has only recently been explored. Several studies have demonstrated that elevated circulating levels of Ang‐II are associated with limb and diaphragm muscle atrophy in recent years [8, 18, 19, 20, 21]. However, changes in RAS metabolism have recently been demonstrated in PWH. Asante et al. [22] demonstrated that circulating levels of Ang‐II were 3.5‐fold higher in PWH compared to people without HIV. These authors also demonstrated that elevated Ang‐II was associated with increased circulating levels of TNF‐α and the elevated intima‐media thickness of the right common carotid artery, a carotid atherosclerotic vascular disease marker. Notably, Deminice et al. [12] demonstrated that human and rodent skeletal muscle express the AT1R receptor. This is a key piece of evidence demonstrating that Ang‐II‐stimulated muscle atrophy can occur by activating AT1Rs on the sarcolemma. In addition, some authors also demonstrated that treatment with the AT1R blocker losartan mitigates diaphragm atrophy and dysfunction induced by mechanical ventilation in rodents [23, 24] and limb muscle atrophy induced by hindlimb immobilization [19]. These discoveries provide the foundation for an improved understanding of the mechanisms responsible for Ang‐II‐induced skeletal muscle atrophy.

In this study, we showed that both PWH using AT1R blockers and ACE inhibitors demonstrated lower plasma Ang‐II levels compared to PWH not using any antihypertensive drugs. However, PWH using AT1R blockers, but not those how use ACE inhibitors, exhibited increased lower limb strength and physical function, lower prevalence of sarcopenia, and decreased oxidative stress. These findings suggest that activation of AT1R, and not only the presence of elevated circulating Ang‐II, appears to be a crucial factor linking RAS to the development of sarcopenia in PWH. Indeed, we demonstrated that PWH using AT1R blockers presented reduced skeletal muscle AT1R expression compared to PWH not using any antihypertensive drugs, suggesting that AT1R activation occurs in the skeletal muscle of PWH and that it is linked to muscle weakness and sarcopenia in this population. This is relevant considering that other mechanisms have been proposed linking elevated Ang‐II and skeletal muscle atrophy.

One of the mechanisms linking elevated Ang‐II and skeletal muscle atrophy is the elevated levels of IL‐6, which has been demonstrated to increase due to Ang‐II [6]. Luther et al. [6] demonstrated that a 3‐h infusion of Ang‐II promoted increased serum IL‐6 concentrations in humans. This is relevant given that IL‐6 can directly promote skeletal muscle atrophy [25]. Haddad et al. [25] demonstrated that IL‐6 infusion in healthy rats resulted in muscle atrophy characterized by a preferential loss of myofibrillar protein (−17). In addition, Zhang et al. [26] proposed that IL‐6 and mediated Ang‐II induced muscle wasting. In addition to IL‐6, studies show that PWH have high levels of other inflammatory interleukins like TNF‐α, IL‐1b, and IFN‐y [27, 28, 29], all that contribute to muscle wasting. However, in the current study, plasma IL‐6 and TNF‐α were not different between PWH using AT1R blockers and ACE inhibitors, suggesting that these inflammatory markers, at least in parts, are not associated with the protection found in PWH using AT1R blockers.

Activation of the “nonclassical” RAS through the increased levels of angiotensin peptides such as Ang(1–7) has also been demonstrated to have a role in muscle wasting [30]. The nonclassical arm of the RAS works in opposition to the effects of the classical pathway (Ang‐II/AT1R). This counteractive mechanism operates through the interaction of Ang1–7 with the G‐protein‐coupled Mas receptor (MasR), which has been linked to systemic anti‐inflammatory and vasodilatory responses when activated [31]. Recently, studies have demonstrated that Ang1–7 protects locomotor muscles against atrophy due to prolonged inactivity or high circulating levels of Ang‐II [32, 33]. More recently, Yoshihara et al. [34] demonstrated that infusion of Ang1–7 protects against mechanical ventilation‐inducing diaphragmatic mitochondrial dysfunction, oxidative stress, and diaphragm weakness. In our study, however, we observed no significant alterations in the circulating levels of Ang1–7 in all groups. Comparable findings have also been reported in other human studies, indicating that neither enalapril (ACE inhibitor) nor losartan (AT1R blocker) resulted in significant changes in plasma Ang1–7 levels [35, 36]. Although we do not have the skeletal muscle level of MasR, our results suggest that Ang1–7 does not play a role in the protective effect of AT1R blockers found in PWH.

Thus, the more plausible mechanism that connects Ang‐II to muscle atrophy in PWH seems to be the direct impact of AT1R signaling on the production of ROS in muscle fibers. We demonstrated that PWH using AT1R blockers, but not those using ACE inhibitors, presented reduced plasma lipid and protein oxidation markers when compared to PWH not using antihypertensive drugs. Also, PWH using AT1R blockers presented lower skeletal muscle 4‐HNE expression compared to the other groups. This is following previous studies demonstrating losartan, but not enalapril, to protect against muscle atrophy and strength loss in preclinical models [24]. The key point in AT1R activation associated with ROS production and oxidative stress is the activation of NADPH oxidase [12]. AT1R activation by Ang‐II promotes increases in NADPH oxidase activity and elevated mitochondrial ROS production leading to speculation that NADPH oxidase/mitochondrial crosstalk exists in skeletal muscle exposed to high levels of Ang‐II [8, 9, 24]. Indeed, unabated ROS production and oxidative stress are key triggers of proteolysis pathways in skeletal muscle via UPS, the major proteolytic system mechanism described in skeletal muscle atrophy associated with chronic diseases [37]. In UPS, muscle‐specific E3 ubiquitin ligase muscle ring finger protein‐1 (MuRF‐1) and MAFbx/atrogin‐1 play a key role as the master regulator of muscle atrophy [38]. Specifically, MuRF‐1 is purported to play a larger role in the ubiquitination of thick filament components such as myosin heavy chain isoforms [39]. Indeed, studies have demonstrated that UPS and their specific skeletal muscle E3 ligases are very sensible from ROS formation [10, 11]. Li et al. [10] demonstrated that adding H_2_O_2_ in myotubes increases the upregulated expression of specific E3 proteins that are thought to regulate muscle catabolism, including atrogin1/MAFbx, MuRF1. Conversely, treatment with antioxidants mitigated ROS production and the expression of MuRF1 and atrogin1/MAFbx, resulting in an enlargement of myotube diameter [11]. Our findings demonstrated that PWH using AT1R blockers exhibited lower skeletal muscle expression of MuRF1 compared to those not employing antihypertensive drugs. These results highlight the role of Ang‐II/AT1R activation in the oxidative stress‐triggered activation of the MuRF1 signaling pathway linked to sarcopenia in PWH. Consequently, our study introduces a novel potential factor contributing to oxidative stress and inflammation induced by HIV and ART—the active axis Ang‐II/AT1R.

This study presents some limitations that must be considered. The absence of an HIV‐negative control group is probably our main limitation, which hinders our ability to quantify the extent to which the amount of Ang‐II in PWH differs and how HIV itself is associated with both RAS and the development of sarcopenia. In addition, this study follows a case–control design rather than a randomized controlled study, which would provide the necessary framework to establish a cause‐and‐effect relationship. Some strengths also may be considered. This study is the first to include PWH in investigating RAS metabolism. This is significant because PWH present a longer life expectancy and multimorbidity, increasing the risk of musculoskeletal impairments such as sarcopenia. Another important aspect of this study is its mechanistic approach involving muscle samples to measure key local oxidative stress and proteolysis markers; acquiring muscle tissue involves an invasive procedure and studies employing this methodology are scarce.

## 5 Conclusion

In conclusion, our results demonstrated that AT1R blockage, but not ACE inhibitors, may protect PWH against muscle wasting, strength loss, and sarcopenia. This suggests that skeletal muscle AT1R activation by Ang‐II is central to RAS metabolism‐induced muscle dysfunction in PWH. Our study adds RAS metabolism activation as a new potential cause of HIV and ART‐related skeletal muscle disorder.

## Author Contributions

Rafael Deminice and Vitor Hugo Fernando de Oliveir designed the study. Rafael Deminice, Ana Lúcia Borsari, and Vitor Hugo Fernando de Oliveir curated and managed the data. Paola Sanches Cella and Camila S. Padilha provided essential laboratory oversight. Rafael Deminice and Paola Sanches Cella conducted the data analyses. Rafael Deminice, Paola Sanches Cella, Ana Lúcia Borsari, Camila S. Padilha, and Vitor Hugo Fernando de Oliveir provided essential subject matter expertise to the design, analysis, and interpretation of the research. Rafael Deminice, Paola Sanches Cella, Camila S. Padilha, and Vitor Hugo Fernando de Oliveir wrote the original draft of the paper. All authors have read and approved of the final manuscript.

## Ethics Statement

The study protocol was approved by the Ethics Board Committee for Research Involving Human Subjects of Londrina State University (#2.305.624) and followed the ethical guidelines of the Helsinki Declaration and Brazilian law. All study participants were volunteers and did not receive any kind of compensation. Informed consent was obtained from the patients or their legal representative.

## Conflicts of Interest

The authors declare no conflicts of interest.

## Declaration of Generative AI and AI‐Assisted Technologies in the Writing Process

During the preparation of this work, authors did not use generative AI and AI‐assisted technologies in the writing process.

## Data Availability

Data are available upon reasonable request.
